# Basomedial amygdala activity in mice reflects specific and general aversion uncontrollability

**DOI:** 10.1111/ejn.15090

**Published:** 2021-01-09

**Authors:** Christian Ineichen, Alexandra Greter, Mischa Baer, Hannes Sigrist, Eva Sautter, Yaroslav Sych, Fritjof Helmchen, Christopher R. Pryce

**Affiliations:** ^1^ Preclinical Laboratory for Translational Research into Affective Disorders (PLaTRAD) Department of Psychiatry, Psychotherapy and Psychosomatics University of Zurich Zurich Switzerland; ^2^ TSE Systems GmbH Bad Homburg Germany; ^3^ Brain Research Institute University of Zurich Zurich Switzerland; ^4^ Neuroscience Center Zurich Zurich Switzerland

**Keywords:** aversion control, basomedial amygdala, chronic social stress, fibre photometry, GCaMP6, learned helplessness effect

## Abstract

Learning adaptive behaviour to control aversion is a major brain function. Detecting the absence of control is also important, although chronic uncontrollable aversion can impact maladaptively on stimulus processing in general. The mouse basomedial amygdala (BMA) contributes to aversion processing with high BMA activity associated with active behavioural responding. The overall aim of the present study was to investigate the associations between aversion (un)controllability, BMA activity and behaviour. Fibre photometry of GCaMP6‐expressing BMA neuron populations was applied in freely behaving adult male mice during exposure to mild electrical shocks, and effects of specific or general (un)controllability were investigated. In a discrete learned helplessness (LH) effect paradigm, mice underwent discrete sessions of pre‐exposure to either escapable shock (ES) or inescapable shock (IES) followed by an escape test. IES mice acquired fewer escape attempts than ES mice, and this co‐occurred with higher aversion‐related BMA activity in the IES group. After 30 days, ES and IES mice were allocated equally to either chronic social stress (CSS)—exposure to continuous uncontrollable social aversion—or control handling (CON), and on days 5 and 15 underwent an IES session. CSS mice made fewer escape attempts than CON mice, and this was now associated with lower aversion‐related BMA activity in the CSS group. These findings suggest that mouse BMA activity is higher when discrete aversion is uncontrollable but becomes lower following chronic uncontrollable aversion exposure. Therefore, BMA activity could be a neural marker of adaptive and maladaptive states consequent to specific and general uncontrollability, respectively.

## Graphical abstract

Ineichen et al. use fibre photometry to measure basomedial amygdala activity to e‐shock. Mice receiving inescapable e‐shock had an escape deficit and higher BMA activity compared to mice receiving escapable e‐shock. Mice undergoing chronic uncontrollable social stress made fewer escape attempts but now had lower BMA activity compared to controls. BMA activity appears to provide a neural marker of adaptive and maladaptive states consequent to specific and general uncontrollability, respectively.

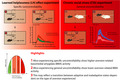

**Abbreviations**
5‐HT: serotonin
a.u.: arbitrary units
ACC: anterior cingulate cortex
BL/6: C57BL/6J
BMA: basomedial nucleus of the amygdala
CON: control handling
CS: conditioned stimulus
CSS: chronic social stress
DRN: dorsal raphe nucleus
EPM: elevated plus maze
ES: escapable shock
e‐shock: electrical shock
fMRI: functional magnetic resonance imaging
IES: inescapable shock
ITI: inter‐trial interval
LH: learned helplessness
MDD: major depressive disorder
PE session: pre‐exposure session
PFA: paraformaldehyde
PFC: prefrontal cortex
vmPFC: ventromedial PFC

## 1 INTRODUCTION

Environmental (un)controllability is a concept of major importance at the interfaces of behavioural neurobiology, psychology, and psychiatry. It concerns the actual or perceived extent to which an individual is able to control an event/stimulus through operant behaviour (Beck & Steer, 1988; Pryce et al., 2011; Seligman et al., 1971). Whilst the concept certainly also applies to reward stimuli it is of most importance with respect to control of aversive stimuli, as achieved through avoidance or escape behaviour. In various mammalian species, e.g., mouse, rat and human, the so‐called learned helplessness (LH) effect paradigm has enabled the objective experimental study of aversion (un)controllability (Maier & Seligman, 1976). The LH effect refers to the paradigm in which experiencing discrete aversive stimuli as uncontrollable, i.e., absence of any possibility to avoid or escape, can lead to the individual responding to subsequent events as if they were also uncontrollable when, in fact, they are not. In the pre‐exposure phase, subjects are allocated at random to the operant conditions of controllable aversion (e.g., escapable electrical shock, e‐shock) versus uncontrollable aversion (e.g., inescapable e‐shock). Critically, the latency to escape e‐shock in the controllable subjects provides the exact duration of the inescapable e‐shock applied to the uncontrollable subjects; that is, both groups experience the exact same amount of aversion and it differs in controllability specifically. In the test phase, all subjects undergo a controllable aversion test (e.g., escapable e‐shock). Subjects that experienced inescapable aversion display a deficit in operant escape behaviour (less escape responses, higher mean latency to escape) relative to those that experienced escapable aversion (Landgraf et al., 2015; Maier & Seligman, 1976; Pryce et al., 2011, 2012).

In animal studies of aversion processing, the LH effect paradigm is informative because it measures aversion uncontrollability, specifically and unequivocally. This is in contrast to paradigms such as the forced swim test and tail suspension test, which are repeatedly claimed to measure “helplessness,” “despair” or “passive coping” but without objective justification (Landgraf et al., 2015; Pryce et al., 2011, 2012). Importantly, the LH effect does not reflect any of these states either. Rather, it constitutes adaptive learning of discrete aversion uncontrollability. Indeed, the LH effect is temporary and after adequate escape test trials, the inescapable group will acquire operant escape behaviour equivalent to that of the escapable group (Maier, 2001). This differs fundamentally from the state of generalised uncontrollability or helplessness, where experiencing chronic aversion uncontrollability can lead to a neurobehavioural state in which unrelated aversive events that are controllable are perceived as uncontrollable (Pryce et al., 2011). In humans, generalised helplessness is a neuropsychological model of depression (Abramson et al., 1989; Disner et al., 2011). Just as the discrete LH effect is a valid (back‐)translational paradigm, it is also possible to develop animal models of generalised uncontrollability, although only a small number have been reported. Studied typically in rodents, chronic social stress (CSS) involves continuous distal exposure of males to larger, dominant, and aggressive conspecific males interspersed daily with brief proximate exposures resulting in attack (Pryce & Fuchs, 2017). During the proximate exposures always and frequently during distal exposure also, the CSS subjects are submissive (e.g., vocalisation, upright defensive posture) but this does not defer attack. Therefore, CSS subjects experience social aversion uncontrollability (Azzinnari et al., 2014; Pryce & Fuchs, 2017). When they are subsequently tested for operant e‐shock responding, CSS mice display deficient avoidance‐escape behaviour relative to controls, thereby providing a mouse model of generalised (social to somatosensory) uncontrollability (Azzinnari et al., 2014). The discrete LH effect and generalised uncontrollability are underlain by behavioural changes related to emotionality, e.g., increased jumping and rearing in intervals between e‐shocks, motivation, e.g., increased response latency to and decreased motor activity during e‐shock, and cognition, e.g., shallow learning curve during escape test trials (Maier & Seligman, 1976; Pryce et al., 2011, 2012).

Current understanding of the neural circuitry underlying aversion (un)controllability has been informed by human studies using electroencephalography, regional cerebral blood flow positron emission tomography, and BOLD‐based functional magnetic resonance imaging (fMRI; Disner et al., 2011), and rodent studies using brain region‐specific lesioning and recording. For example, in healthy humans studied using fMRI, pain perceived as uncontrollable induced greater BOLD signal in the anterior cingulate cortex (ACC) compared to pain perceived as controllable (Salomons et al., 2004). In subjects with major depressive disorder (MDD), the anticipation of uncontrollable pain induced greater BOLD signal in ACC and amygdala as compared with healthy controls, and amygdala BOLD signal correlated positively with helplessness in the MDD subjects specifically (Strigo et al., 2008). In rats, c‐Fos immediate‐early‐gene protein immunostaining revealed that inescapable e‐shocks result in more neuronal activation in amygdala, hypothalamus, locus coeruleus, and dorsal raphe nucleus than do escapable e‐shocks (Liu et al., 2009). Processing (un)controllability requires the integration of somatosensory aversion onset/offset with somatomotor feedback from operant behaviour (e.g., escape responses; Robbins, 2005). The prefrontal cortex (PFC) is the primary region of such sensory‐motor integration, and the rodent ventromedial PFC (vmPFC) is the analogue of the primate ACC (Laubach et al., 2018). In rats, pharmacological excitation of the vmPFC prior to inescapable e‐shocks blocked the LH effect at the escape test phase (Amat et al., 2008). Vice versa, pharmacological inhibition of the vmPFC prior to escapable e‐shocks resulted in rats behaving at the escape test phase as if they had been inescapable (Amat et al., 2005). One projection region of the rodent vmPFC is the basomedial nucleus of the amygdala (BMA; Adhikari et al., 2015). In mice, electrophysiology and optogenetics have been used to demonstrate that: (a) different BMA neuron populations are selectively active in aversive or safe environments, (b) activation of BMA neurons decreases defensive freezing behaviour, and (c) vmPFC‐BMA long‐range projections mediate the top‐down inhibition of defensive freezing behaviour (Adhikari et al., 2015).

Building on this evidence for an important role of the BMA in aversion processing and potential involvement in circuits underlying (un)controllability processing, in the present study we applied fibre photometry to investigate whether BMA activation is sensitive to the prevailing (un)controllability of the aversive environment. In a first experiment, BMA activity was studied in a discrete LH effect paradigm (Pryce et al., 2012). Mice that experienced inescapable e‐shocks made fewer escape attempts than did escapable e‐shock mice, and this was associated with higher e‐shock‐related BMA activity in the former group. In a second experiment, these same mice were allocated in a counterbalanced manner to CSS and control conditions (Azzinnari et al., 2014) and underwent sessions of inescapable e‐shocks. Because the LH effect is a less stressful procedure compared with CSS, we conducted this first in order to minimise the carryover effect from one experiment to the other. Whilst the mice that experienced chronic social aversion made fewer e‐shock escape attempts than did control mice, this was now associated with lower e‐shock‐related BMA activity in the CSS mice. Therefore, in an otherwise non‐aversive environment, BMA activity is higher to uncontrollable than controllable aversion, and thereby possibly contributes to adaptive re‐learning when the aversion becomes controllable. However, in a chronically aversive environment, BMA activity to an unrelated and uncontrollable aversion is reduced, and thereby possibly contributes to the maladaptive generalisation of uncontrollability.

## 2 MATERIALS AND METHODS

### 2.1 Animals and ethical standards

The study was conducted under a permit for animal experimentation (ZH 155/2018) issued by the Veterinary Office of Canton Zurich. Male C57BL/6J (BL/6) mice were bred in‐house and weaned at age 3 weeks, when they were caged together in littermate pairs. At experimental onset, mice were aged 15 weeks and weighed 30–32 g. In the chronic social stress experiment, the resident mice were males of the CD‐1 strain (Janvier, Le Genest‐Saint‐Isle, France). They were aged 8–9 months and ex‐breeders, weighing 38–50 g and caged singly when not in the experiment. Cages were type 2L (33 × 21 × 14 cm), individually ventilated at 22–24°C and 50%–60% humidity and contained sawdust, paper tissue as nesting material and a sleeping shelter. Mice were maintained on a reversed 12:12 hr light‐dark cycle with lights off at 07:00–19:00 hr. They were provided with ad libitum standard complete‐pellet diet (Kliba Nafag, Granovit) and water was also available ad libitum.

### 2.2 Study design

At study onset, the BL/6 mice (*N* = 24) were handled for 5 min on each of 3 days. They then underwent stereotactic surgery required for fibre photometry, followed by a 20‐day period of recovery and viral vector transduction and expression. The mice were then studied in the learned helplessness effect experiment which involved four daily behaviour‐photometry test sessions over 5 days. After a 30‐day period during which mice were undisturbed except for cage changing, they were then studied in the chronic social stress—inescapable e‐shock experiment comprising a 15‐day period of either CSS or control handling and behaviour‐photometry test sessions on days 5 and 15. After the completion of this experiment, all mice were perfused for brain histological assessment.

### 2.3 Apparatus

#### 2.3.1 Behavioural testing

Electrical shock (e‐shock) presentation and behavioural measurement were conducted using a purpose‐built two‐way system (Multi Conditioning System, Version 1.0; TSE Systems GmbH). The e‐shock grid floor measured 18 (L) × 28 (W) cm and comprised 32 stainless steel rods (Ø = 10 mm, inter‐rod centre‐to‐centre distance = 4 mm). A rectangular arena (14 (L) × 28 (W) × 25 (H) cm) made from transparent Plexiglas and without floor or ceiling was positioned on top of the grid. A vertical indent (width 5 mm) running from top to bottom and at the mid‐point of the front and back walls of the arena provided the only landmarks dividing the arena into identical “left” and “right” compartments (i.e., there was no hurdle or tunnel). Two transparent Plexiglas lids, separated by a narrow gap for passage of the photometry fibre patch cord (see Section 2.3.2), were placed on top of the arena, and a metal waste tray was positioned underneath the grid floor. A metal frame surrounding the grid and arena was fitted with infrared light‐beam sensors for object‐detection along its front and back edges; this detected mouse position and movement in the horizontal plane along the width of the arena including compartment transfers. The sensors were spaced 14 mm apart, except at the mid‐point where the distance between the left‐side sensor and right‐side sensor was 28 mm. This apparatus was contained within an attenuating chamber equipped with a ventilation fan, house lights set to provide 8 lux in the arena, a loudspeaker emitting low‐level white noise, and a concave lens peephole for observation. An opening in the ceiling allowed for the fitting of a peephole camera (Logitech) which allowed for video recording of the test sessions on a PC running the LabVIEW software. The behavioural test unit was interfaced with a control unit which was connected to a PC running hardware‐control and data‐collection software. The single‐system input was e‐shock. The infra‐red sensor system was programmed to discriminate the left and right compartments and allowed for movement detection within each compartment and transfer between compartments based on sensor beam breaks, during e‐shock trials and inter‐trial intervals; these were the system outputs. The onset of e‐shock was time‐stamped onto the photometry data record via a TTL interface.

#### 2.3.2 Fibre photometry and stereotactic surgery for rAAV vector infusion and optic fibre implantation

**FIGURE 1:**
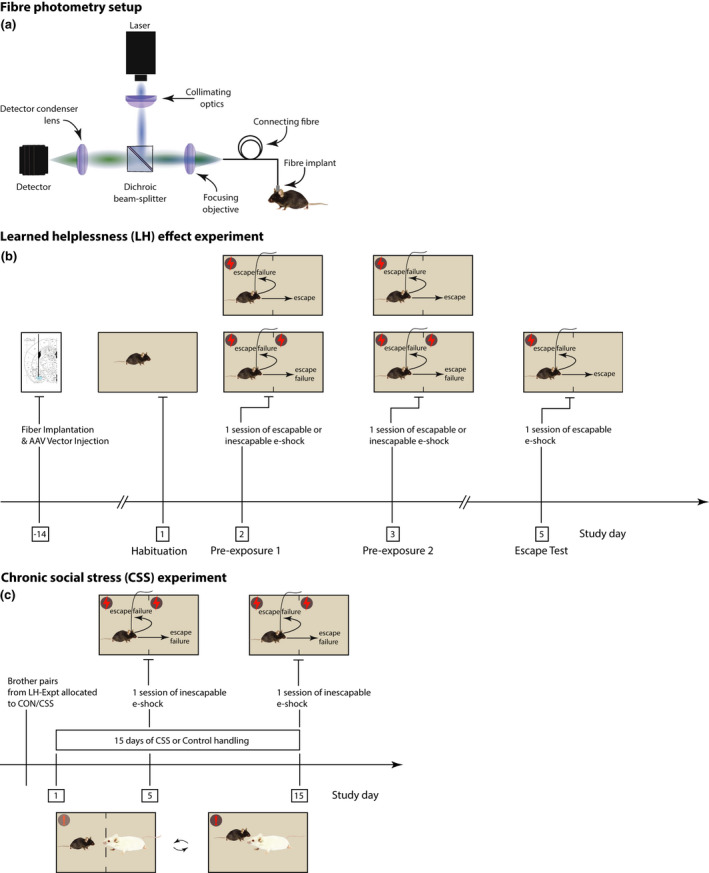
(a) Schematic of the fibre photometry setup. (b) Experimental design for the learned helplessness (LH) effect experiment. Naive mice in littermate pairs were handled and underwent surgery for rAAV GCaMP6 vector infusion and chronic fibre implantation. Following 14‐day recovery, mice were placed in the arena for a habituation session, with activity scores used to counter‐balance allocation of one littermate to the escapable e‐shock (ES) group and the other littermate to the inescapable e‐shock (IES) group. Mice then underwent 2 daily ES or IES pre‐exposure sessions, with the IES littermate yoked to the ES littermate in terms of e‐shock duration. Two days later, all mice underwent an ES escape test. In the pre‐exposure sessions and the escape test, fibre photometry of basomedial amygdala (BMA) activity was conducted throughout the session. (c) Experimental design for the chronic social stress‐inescapable e‐shock experiment. Thirty days after the completion of the LH effect experiment, ES‐IES littermate pairs were allocated to either chronic social stress (CSS) or control handling (CON), counterbalancing on the number of escape responses in the escape test. Mice then underwent the 15‐day CSS/CON procedure. On day 5 and day 15 all mice underwent an IES session with 3‐s e‐shocks during which fibre photometry of BMA activity was conducted throughout the session

Fibre photometry was used for optical recording of neural activity in the BMA in freely moving mice (Lütcke et al., 2010; Sych et al., 2019) (Figure 1a). Briefly, fibre photometry uses a single‐fibre optic device to detect neural activity‐dependent fluorescence emission of specific proteins. It requires an excitation light source (typically a laser or LED), a high‐sensitivity photodetector, and customised software for signal processing. An optic fibre is implanted in the brain region of interest. At the cranium surface, the fibre is connected to a flexible patch‐cord that in turn connects the implant to the optical setup. Typically, the same flexible patch‐cord is used for delivery of excitation light and collection of fluorescence emission. The brain region of interest is infused with a viral vector expressing a genetically encoded, calcium activity‐dependent fluorescent protein, e.g., GCaMP6 (Schlegel et al., 2018). In this study, the viral vector was AAV2.9‐hSyn‐GCaMP6m. Given that in the BMA the majority of neurons are spiny glutamatergic neurons (~80%) and the minority are GABA interneurons (~20%; Duvarci & Pare, 2014), and given that the probability of transduction‐expression of AAV vectors driven by the hSyn promoter and administered at high titres is similar in glutamate neurons and GABA neurons in mixed populations (Nathanson et al., 2009), then spiny glutamatergic neurons would be expected to dominate the fibre photometry signal in the BMA.

Both mice per littermate pair were operated on the same day, either both in the left hemisphere or both in the right hemisphere, with alternation between successive littermate pairs. Surgery was conducted using a stereotactic system (Angle Two™; Leica). Mice received buprenorphine 30–60 min pre‐operatively (Temgesic, 0.1 mg/kg s.c.). Anaesthesia induction was conducted using 4% isoflurane (in pure oxygen) followed by transfer of the mouse to a warming pad and placement in a nose cone for delivery of 1.50%–1.75% isoflurane in oxygen. A precision vaporiser and a waste gas absorption unit were used. Body temperature was maintained at 37°C. The absence of pain reflex responses was controlled for, mucous membrane colour and respiratory rate were observed throughout, and capillary refill time was checked as necessary. The cranium was shaved and fixed in a stereotactic head frame, with lidocaine applied to the ears prior to the insertion of ear bars (EMLA Creme 5%, Aspen Pharma) and ophthalmic ointment applied to the eyes (Viscotears, Novartis). A skin incision was made at the cranium midline, connective tissue was removed, and a hole was drilled through the cranium to prepare the single injection site. A 10 μl glass syringe fitted with a 33G bevelled stainless‐steel needle (NanoFil Syringe; World Precision Instruments) was attached to an ultra‐micro pump (UMP3, Micro4; World Precision Instruments). The syringe contained the viral vector AAV2.9‐hSyn‐GCaMP6m (qtitre 5.8 × 10^13^ GC/ml), which was infused into the dorsal BMA in a volume of 300 nl at a flow rate of 50 nl/min. The stereotactic coordinates used were bregma AP −1.06, ML ± 2.5, DV −5.35 which, as established in pilot surgeries, yielded viral infusion at AP −1.34, ML ± 2.5, DV −5.35, according to a mouse brain atlas (Franklin & Paxinos, 2008). After the completion of injection, the needle remained in place for 5 min to allow focal diffusion and reduce reflux and was then withdrawn slowly. Next, an optic fibre implant (Ø = 200 µm, Thorlabs) was inserted and extended down to DV −5.2 mm (Franklin & Paxinos, 2008). The dorsal extent of the fibre was embedded in a ceramic ferrule (Ø = 2.5 mm), which was cemented to the cranium surface. Chronic adhesion to the cranium was achieved using iBond (Kulzer, Total Etch) and a thin ring of Charisma (Kulzer), which were cured with UV light. Dental cement (Tetric EvoFlow) was applied to the cranium and around the implant, followed by curing with UV light. To facilitate head fixation for patch‐cord connection at behavioural testing, a tungsten head post was cemented posterior to the implant. Total weight of fibre implant, head post, and cement measured <1 g. Post‐operative analgesia comprised buprenorphine injection at 4–6 and 8–10 hr after initial injection and buprenorphine in drinking water on post‐operative days 1–3. Mice were controlled (surgical site, body weight, physical status, behaviour) on post‐operative days 1–10.

To provide excitation light a 488 nm laser (OBIS 488 LX; Coherent Inc.) was used. The laser was modulated at 970 Hz and run at 80% maximal output power. Laser light was attenuated so that 640–3200 μW per mm^2^ were delivered at the optic fibre tip. The light was directed through an excitation filter, reflected off a dichroic mirror (MDF‐GFP2 filter set, Thorlabs) and, using an objective (F240FC‐A, Thorlabs), focussed into a fibre patch cord (Ø = 200 µm UM22‐200, Thorlabs, NA 0.22; Figure 1a). The flexible patch‐cord ran through a central hole in the roof of the attenuation chamber and was connected to the optic‐fibre ferrule on the mouse cranium via a ceramic sheath. This allowed the mouse to move freely during behavioural testing. GCaMP6 fluorescence was back‐propagated through the patch‐cord, collimated by the objective, separated spectrally from the excitation light by the dichroic mirror and an emission filter set (MDF‐GFP2), and focused on a photomultiplier detector (PMM02, Thorlabs) using a condenser lens (ACL2520U, Thorlabs). To further increase detection sensitivity and decrease instrumental noise, a lock‐in detection scheme was used based on 970 Hz modulation: raw data were acquired at 2 kHz, digitised using a DAQ board (NI USB‐6211; National Instruments), and the fluorescence intensity was extracted at an actual sampling rate of 20 Hz. This was sufficient to capture the relatively slow kinetics of the Ca^2+^‐dependent signal (Chen et al., 2013). Custom‐written software code was used for data acquisition (LabVIEW, 2020; see Section 2.4.4).

### 2.4 Aversive stimulus exposure and behavioural testing

#### 2.4.1 Learned helplessness effect

To investigate the effects of e‐shock (un)controllability on behaviour and BMA activity, a previously described LH effect paradigm was used (Pryce et al., 2012). For each session, the mouse was weighed and placed in the arena on the grid floor (see Section 2.3). After each session, the arena was cleaned and then wiped with 70% ethanol for the next mouse. The paradigm was conducted as follows (Figure 1b).

Day 1, Habituation. Mice were placed singly in the arena for 15 min without any e‐shock. The measure of interest was the total distance moved based on beam breaks (arbitrary units, a.u.). Based on these values, in each littermate pair one mouse was allocated to the escapable e‐shock (ES) group and the other to the inescapable e‐shock (IES) group, such that the mean distance moved was similar in the two groups (ES: 58,151 ± 3,415 a.u., IES: 60,117 ± 2,421 a. u., *p* = .74).

For each of the following sessions, after weighing the mouse, the optic fibre was connected to the patch cord and the mouse was placed in the arena on the grid floor and the session started.

Days 2 and 3, E‐shock Pre‐exposure Sessions 1 and 2 (hereafter referred to as Pre‐exposure Sessions 1 and 2). The mouse allocated to the ES group was placed in the arena and exposed to 30 trials of mild e‐shock. The maximum duration of the e‐shock was 6 s and at 0–1 s had an amplitude of 0.10 mA and thereafter of 0.15 mA. The initial amplitude of 0.1 mA was used as a cue to “announce” the e‐shock and thereby reduced e‐shock jumping responses (Pryce et al., 2012). The inter‐trial interval (ITI) between successive e‐shocks was fixed at 50 s. For escapable e‐shock (ES) mice, regardless of which compartment the mouse was in at e‐shock onset, the first transfer between compartments—indicated by the change in object location detected by the infrared‐light beam sensors—resulted in immediate e‐shock termination, i.e., escape. The subsequent ITI was then initiated. The escape latencies of the ES mouse were saved and then used to provide the e‐shock durations to be applied to the littermate in the inescapable e‐shock (IES) group. That is, the ES and IES mice in a littermate pair received the same number and duration of e‐shocks and in the same order. This procedure was repeated on day 3. The measures of interest were: the number of e‐shock transfers, i.e., escape responses in ES mice and “escape attempts” in IES mice; mean e‐shock duration, i.e., mean escape latency in ES mice and inescapable e‐shock duration in IES mice; mean e‐shock distance moved per second (velocity, a.u./s). In addition, ITI % time freezing was measured from video images (see below).

Day 5, Escape test. After a 2‐day period during which littermate pairs remained undisturbed, all mice were exposed to 30 trials of escapable e‐shock (ES) with a maximum duration of 3 s at an amplitude of 0.10 mA at 0–1 s and 0.15 mA at 1–3 s, with a fixed ITI of 50 s. Now the e‐shock duration received by both ES and IES mice was dependent on their own e‐shock behaviour (escape, escape failure). The measures of interest were: the number of e‐shock transfers (escape responses); mean e‐shock duration (escape latency), mean e‐shock distance moved per second, and ITI % time freezing.

For analysis of ITI % time freezing, video recordings of the test sessions were analysed with an open‐source video analysis pipeline (ezTrack, Pennington et al., 2019). The following parameters were used: an individual motion cut‐off output (mt cutoff) to distinguish between pixel changes attributable to mouse movement and random fluctuations; a constant cut‐off set at nine frames (MinDuration = 9) had a duration of 1 s and defined the minimum frame number across which the signal needed to remain unchanged for the scoring of a freezing episode; a cut‐off constant set at 350 pixels (FreezeThresh = 350) defined the maximum number of frame‐to‐frame pixel changes tolerated for the mouse to continue to be scored as freezing. The values selected for the latter two parameters were used because they maximised the agreement between direct experimenter scoring and script‐based scoring of freezing behaviour, in three video sessions selected at random.

#### 2.4.2 Chronic social stress and inescapable e‐shock exposure

After the completion of the LH effect experiment and an interval of 30 days, the ES‐IES littermate pairs were allocated to either the control (CON) or CSS group, counter‐balancing the groups according to the number of escape responses in the escape test whilst also ensuring that the e‐shock distance moved per second and the mean BMA Ca^2+^ activity profiles in the escape test were similar in mice allocated to the CON and CSS groups. The standard CSS procedure is described in detail elsewhere (Azzinnari et al., 2014; Figure 1c). Briefly, the home cages of resident CD‐1 mice were separated longitudinally into two equal compartments using transparent, perforated Plexiglas dividers. On CSS/CON day 1, the BL/6 (ES‐IES) littermate pairs allocated to the CSS group were separated and placed singly in the cages of CD‐1 mice. Behavioural interactions were observed, and the intruder (BL/6) and resident (CD‐1) were maintained together for either 60 s cumulative attack time or 10 min maximum, whichever was reached first. During these attack sessions, in contrast to the standard protocol, the central divider was removed from the cage; this change was introduced to avoid the optic fibre becoming caught in the divider perforations. The divider was then re‐inserted in the cage and the BL/6 and CD‐1 mice were placed in separate compartments and then remained in distal sensory contact for the next 24 hr. On each of days 2–15, the BL/6—CD‐1 mouse pairings were rotated, and the above procedure repeated, such that each CSS BL/6 mouse was placed together with 9–10 different CD‐1 mice in total. To prevent bite wounds the lower incisors of CD‐1 mice were trimmed every third day (Azzinnari et al., 2014). Each BL/6 mouse was observed to be attacked by each CD‐1 mouse with attacks comprising chase, box, and bite behaviours by CD‐1 mice. The overall daily mean duration of attacks was 51.5 ± 2.8 s (± *SEM*); this is typical (e.g., Azzinnari et al., 2014; Carneiro‐Nascimento et al., 2020)) despite the modifications necessitated by optic fibre implants. In addition to attempting to avoid (active, passive) and escape (active) CD‐1 mice, BL/6 mice frequently emitted the submissive behaviours of upright stance and vocalisation. These submissive behaviours did not defer attacks by the CD‐1 mice, such that CSS mice experienced daily defeat by and exposure to an uncontrollable social stressor. The ES‐IES littermate pairs allocated to CON were maintained as pairs and handled and weighed on each of days 1–15.

On day 5 and day 15 of the CSS/CON procedure, all mice underwent a session of exposure to 30 trials of inescapable e‐shock at an amplitude of 0.10 mA at 0–1 s and 0.15 mA at 1–3 s, a fixed ITI of 50 s and with simultaneous fibre photometry recording (Figure 1c). Based on our previous findings using escapable e‐shocks, CSS mice would be expected to make fewer escape responses and have longer escape latencies than CON mice (Azzinnari et al., 2014). Therefore, to ensure that CSS and CON mice were compared in their BMA activity under the same aversion conditions, inescapable e‐shock was used. For behaviour‐photometry sessions, the mouse was weighed, connected to the optic fibre and placed in the arena on the grid floor. After each session, the arena was cleaned and then wiped with 70% ethanol for the next mouse. The behavioural measures of interest were: number of e‐shock transfers, mean e‐shock distance moved per second, and ITI % time freezing.

#### 2.4.3 Histology

**FIGURE 2:**
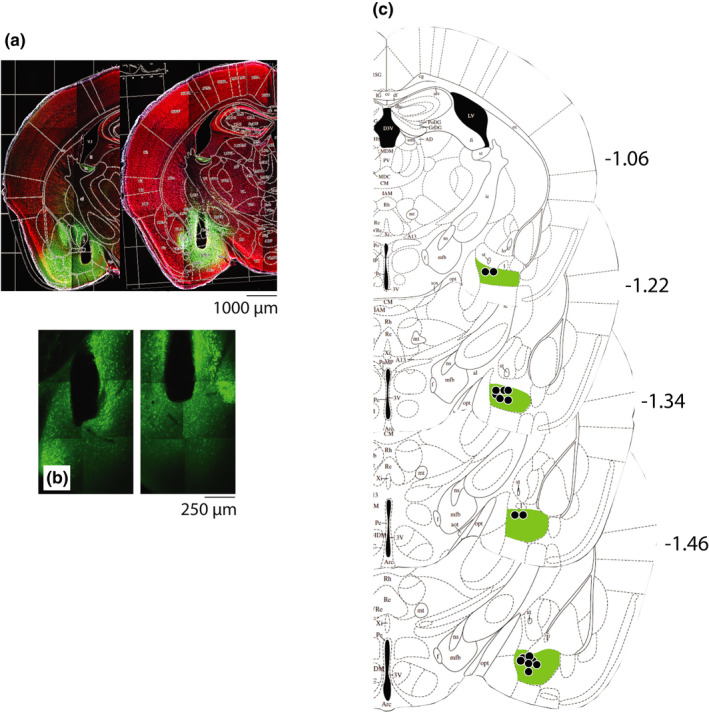
Histological verification. (a) Representative microscope images (5×) of Nissl‐stained coronal brain sections at −1.22/−1.34 mm relative to bregma, showing the location of the optic fibre implant including its tip in the dorsal basomedial amygdala and the colocalised rAAV vector‐expressed GCaMP6 fluorescence. (b) Representative microscope images (20×) of coronal brain sections at bregma −1.22 showing cellular localisation of rAAV vector‐expressed GCaMP6 fluorescence. (c) Diagrams of coronal sections (Franklin & Paxinos, 2008) indicating the estimated location of the optic fibre tip and GCaMP6 expression as estimated from histology. Mouse littermate‐pairs were both injected in the left or right hemisphere, and each experimental group (ES vs. IES and CSS vs. CON) contained a combination of left‐ and right‐hemisphere‐injected mice; for illustration purposes, all location estimates are depicted in the left hemisphere

Following the completion of the study, mice underwent deep anaesthesia with pentobarbital (50 mg/kg i.p.) and transcardial perfusion with phosphate‐buffered saline (PBS, pH 7.4, 20 ml) followed by paraformaldehyde (PFA, 4% in PBS, 60 ml). The head was removed with the fibre implant intact and post‐fixed for 3 days in 4% PFA. The brain was then isolated from the skull and the optic fibre implant removed. The brain was sectioned coronally at 100 μm using a vibratome (Leica). Serial sections were placed free‐floating in 0.1 M PBS, rinsed in PBS, and then underwent Nissl staining (NeuroTrace 640/660 Deep‐Red Fluorescent Nissl Stain; Thermo Fisher), followed by washing in PBS, mounting on microscope slides, addition of Dako/DAPI fluorescence mounting medium (Sigma Aldrich), and cover‐slipping. Using an epifluorescence microscope (Zen 2; Zeiss), mounting medium allowed for the localisation of GCaMP6 expression and Nissl staining for the localisation of the optic fibre placement. Using a mouse brain atlas (Franklin & Paxinos, 2008) the bregma level of the section that included the most ventral position of the fibre tip in the dorsal BMA was identified. Representative examples of the histological verification of GCaMP6 expression and optic fibre tip placement, as well as the estimation of the optic fibre tip placements relative to bregma for all subjects included in the statistical analyses, are given in Figure 2.

#### 2.4.4 Fibre photometry data analysis

Fibre photometry data were analysed with custom‐written MATLAB programmes. To ensure the synchronisation of the optical signals and behavioural data, a TTL signal generated by the behavioural system coincident with e‐shock onset was recorded simultaneously with the photometry signal. Optical signal data were demodulated at 970 Hz and downsampled to a sampling frequency of 20 Hz, thereby excluding some of the broad‐spectrum signal noise. For analysis of aversion‐related BMA activity relative to e‐shock onset, each trial was analysed individually: The 15 s period prior to e‐shock onset was defined as the baseline period for that trial. For each 0.05 s time bin (*t*) after e‐shock onset, the normalised (*z*‐scored) signal intensity (*F*) was calculated using the formula (*F*(*t*) − *F*_0_)/*SD*_0_, where *F*_0_ and *SD*_0_ denote mean and standard deviation fluorescence intensity in the baseline period. Thereafter the mean *z*‐scored *F*(t) for trials 1–30 was calculated for each *t* and each mouse. For statistical analysis, these mean *z*‐scored signal *F*(*t*) values were then binned into 1 s time intervals for seconds 1–12 after e‐shock onset (aversion‐related BMA activity). In addition, using seconds 21–35 after e‐shock onset to represent the ITI without overlap with baseline or aversion‐related activity, ITI BMA activity was calculated using the same baseline and steps as above. In addition, for completeness, the ΔF/F signals were also calculated ((*F*(*t*) − *F*_0_)/*F*_0_), with *F*_0_ again being the mean baseline fluorescence intensity during the 15 s prior to e‐shock onset (see Figures S1 and S2).

### 2.5 Statistical analysis

Of the 24 mice, six were excluded due to optic fibre and/or rAAV vector location outside the region of interest or implant displacement. In the remaining 18 mice, a small number of their sessions contained artefacts in the photometry record that precluded analysis of that specific session only. Therefore, statistical analysis was conducted in the LH effect experiment with data contributed by 7–8 ES and 8–9 IES mice and in the CSS/CON‐IES experiment with the data contributed by 7–8 CON (CON‐ES *N* = 4, CON‐IES *N* = 3–4 mice) and 8 CSS (CSS‐ES *N* = 4, CSS‐IES *N* = 4 mice). Statistical testing was conducted primarily with a general linear model (GLM) for analysis of variance (ANOVA) or with Student's *t* test, either with SPSS (version 25; SPSS Inc.) or the built‐in statistical libraries of Python (5.6.0, Python.org). A first analysis investigated for the effects of the implanted hemisphere; there was no significant effect (*p* ≥ .18) and the main analysis did not take implanted hemisphere into account, therefore. For the LH effect experiment, behavioural measures were analysed by GLM ANOVAs with fixed effects of Group (ES, IES) and Pre‐exposure session (1, 2) and a random effect of mouse ID, and by *t* tests for the escape test. For BMA activity, a first GLM ANOVA analysis was conducted for each session separately with fixed effects of Group (ES, IES) and Time (post‐e‐shock seconds 1–12) and a random effect of mouse ID. A second GLM ANOVA was conducted with Session (pre‐exposure 1, pre‐exposure 2, escape test) added as a fixed effect. For the escape test, in ES and IES mice separately, the correlation between e‐shock escape responses and BMA activity (mean signal at 2–8 s after e‐shock onset) was analysed. For the CSS/CON‐IES experiment, behavioural measures were analysed by GLM ANOVAs with fixed effects of Group (CSS, CON) and Day (5, 15) and a random effect of mouse ID. For BMA activity, a first GLM ANOVA was conducted for each session separately with fixed effects of Group (CSS, CON) and Time (post‐e‐shock seconds 1–12) and a random effect of mouse ID. A second GLM ANOVA was conducted with Day (5, 15) added as a fixed effect. For CSS/CON day 5, the correlation between e‐shock transfer responses and BMA activity (mean signal at 2–8 s after e‐shock onset) was analysed; the analysis was conducted separately for each of the sub‐groups CON‐ES, CON‐IES, CSS‐ES, and CSS‐IES mice. For GLM ANOVAs, in the case of significant main or interaction effects, post hoc testing was conducted using the least significant difference. Statistical significance was set at *p* ≤ .05. All data are presented as mean ± *SEM*. Data graphs were prepared in Python.

## 3 RESULTS

### 3.1 Learned helplessness effect

**FIGURE 3:**
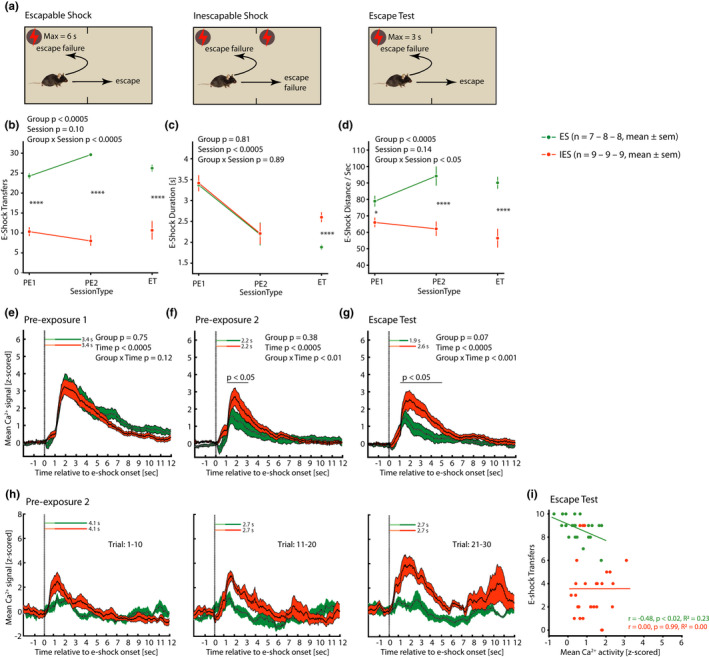
Behaviour and basomedial amygdala (BMA) activity in the learned helplessness effect experiment. (a) Schematic for ES and IES sessions: mice were placed in an arena comprising identical left and right compartments with a grid floor conducting mild electrical shock. (b) Number of e‐shock transfers for the 30 trials in each of pre‐exposure Sessions 1 and 2 (PE1, PE2) and the escape test (ET). For ES mice, these were escape responses in PE1, PE2, and ET, and for IES mice they were “escape attempts” in PE1 and PE2 and escape responses in ET. (c) Mean e‐shock duration as determined by the latency to e‐shock transfer in ES mice in PE1, PE2, and by the latency to e‐shock transfer in ES and IES mice in ET. (d) Mean distance moved per second of e‐shock, which was escapable in PE1, PE2 and ET in ES mice and inescapable in PE1 and PE2 and escapable in ET in IES mice. (e–g) Mean BMA activity in ES and IES mice in the 30 trials in PE1, PE2 and ET, respectively, from 2 s pre‐e‐shock to 12 s post‐e‐shock onset. For the corresponding data expressed as ΔF/F see Figure S1. (h) In a representative ES‐IES littermate pair in session PE2, mean BMA activity in trials 1–10, 11–20, 21–30. (i) Scatterplots for e‐shock transfers versus BMA activity (mean of signal at 2–8 s after e‐shock onset) for ES and IES mice in the escape test, with each mouse contributing data for trials 1–10, 11–20, 21–30. The regression lines are presented together with Pearson's correlation coefficient and *R* ^2^ values. For behavioural data, PE‐session *p*‐values are for GLM ANOVA analysis and post hoc least significant difference testing, and ET *p*‐values are for *t* tests: *p < .05, **p < .01, ***p < .001, *p* < .0005. For fibre photometry data (e–g), *p*‐values are for GLM ANOVA analysis and post hoc least significant difference testing

In LH effect experiments (Figures 1b and 3a), it is important to verify that mice allocated to the ES and IES groups are similar in behaviour prior to the onset of aversion exposure. Accordingly, the behavioural data for pre‐exposure Session 1 were first analysed as six blocks of five trials each: For e‐shock transfers (Figure S3a), the number was similar in IES and ES mice in trials 1–5 and thereafter transfers decreased in IES mice and increased in ES mice such that IES mice made significantly less transfers than ES mice (Group × Block interaction effect: *F*_5,70_ = 6.21, *p* < .0005). For e‐shock distance moved per second (Figure S3b), this was similar in IES and ES mice in trials 1–5, 6–10, and 16–20 and then increased in ES mice specifically and became consistently and significantly lower in IES than ES mice in trials 21–25 and 26–30 (Group × Block interaction effect: *F*_5,70_ = 2.71, *p* < .05). Therefore, mice allocated to the ES and IES groups were similar in aversion‐related behaviour at the onset of the experiment.

For the main analysis of the behavioural data the total or mean score per mouse across all 30 trials was used. Mice underwent two e‐shock pre‐exposure sessions followed by an escape test; the two groups—ES and IES—displayed the expected behavioural differences. Therefore, in the two pre‐exposure sessions, with regards to e‐shock transfers (Figure 3b), mice in the ES group made a transfer (escape) response to the e‐shock in most trials and the mean number of e‐shock transfers was higher in Session 2 than 1. Mice in the IES group made fewer e‐shock transfers (“escape attempts”) than did ES mice and their mean number of transfers was lower in Session 2 than 1. There was a significant Group × Session interaction effect (*F*_1,17.9_ = 20.06, *p* < .0005) and a Group main effect (*F*_1,18.5_ = 314.53, *p* < .0005). That ES mice had learned that the e‐shock was controllable whereas IES mice had learned that it was not, was confirmed by their relative e‐shock transfers in the escape test: in both groups, the number of transfer responses was similar to that at pre‐exposure Session 2, with IES mice making fewer escapes than did ES mice (*t*(15) = 5.87, *p* < .0005), i.e., the discrete LH effect was established. For the measure mean e‐shock duration (Figure 3c), in the pre‐exposure Sessions this was of course identical in ES and IES mice, being determined in both groups by the escape latencies of ES mice. E‐shock durations were shorter in Session 2 than Session 1, indicating that ES mice learned to escape more rapidly (Session main effect: *F*_1,16.8_ = 37.19, *p* < .0005). In the escape test, in accordance with their fewer escapes, e‐shock duration was longer in IES than ES mice (*t*(15) = −5.41, *p* < .0005); relative to pre‐exposure Session 2, mean e‐shock duration increased in IES mice and decreased in ES mice. For e‐shock distance moved per second (Figure 3d), a measure of motivation, in the pre‐exposure Sessions this was shorter in IES than ES mice and whereas the mean value increased in ES mice from Sessions 1 to 2 it decreased in IES mice (Group × Session interaction effect: *F*_1,16.3_ = 7.20, *p* < .02; Group main effect: *F*_1,17.8_ = 21.14, *p* < .0005). Similarly, in the escape test, e‐shock distance per second was shorter in IES than ES mice (*t*(15) = 4.91, *p* < .0005). To summarise the behavioural repertoires of ES and IES mice during e‐shock, whereas ES mice ran forwards and typically crossed the mid‐point of the arena and then slowed down after e‐shock termination, IES mice took up a more rounded body posture during e‐shock and then moved relatively slowly either forwards or backwards within the compartment in which they were located at e‐shock onset and until e‐shock termination. Because of the refinement of using an initial amplitude of 0.1 mA e‐shock at 0–1 s, all mice displayed very few jumping responses to e‐shock. Mouse behaviour during the inter‐trial intervals of each test phase is summarised in Table 1. For the measures total ITI transfers and mean locomotor distance per ITI, behaviour was similar in IES and ES mice, and remained constant across the test phases. Finally, the percentage of time spent freezing was lower in IES than ES mice in Session 2 specifically (*t*(15) = 2.79, *p* < .03). This effect has been observed previously and is associated with preparation for e‐shock escape responding in ES mice specifically (Azzinnari et al., 2014).

**TABLE 1:** Summary table for inter‐trial interval behaviour in the LHE experiment and the chronic social stress‐E‐shock experiment

Parameter		Learned helplessness effect						Chronic social stress‐E‐shock			
		Pre‐exposure 1		Pre‐exposure 2		Escape test		CSS day 5		CSS day 15	
		ES (7)	IES (9)	ES (8)	IES (9)	ES (8)	IES (9)	CON (8)	CSS (8)	CON (8)	CSS (8)
Transfers (all ITIs)	Mean	71.4	96.1	70.8	91.9	116.5	118.8	90.0	125.5	119.1	107.0
	*SEM*	2.9	11.3	5.9	7.0	7.7	9.9	8.3	17.8	13.3	5.7
Locomotion (per ITI; arbitrary units)	Mean	1,700.6	2,109.1	1,650.5	2,049.2	2,000.4	2,195.9	1,620.3	1,872.7	2,080.7	1,761.5
	*SEM*	67.2	155.1	106.5	114.1	88.4	127.3	131.5	78.4	160.6	72.8
% Time freezing (ITI mean)	Mean	18.8	14.7	20.4	11.9^*^	15.9	10.4	14.5	11.0	13.1	16.2
	*SEM*	1.8	2.5	1.9	1.1	2.7	1.6	1.8	1.8	1.8	1.8

* *p* < .05.

The mean general temporal profile of BMA activity in each test session, both in this experiment and the CSS experiment, is exemplified by the data for pre‐exposure Session 1 (Figure 3e): BMA activity was higher in seconds 2–9 after e‐shock onset compared with seconds 1 and 10–12 after e‐shock onset (main effect of Time: *F*_11,154_ = 36.80, *p* < .0005). The 1 s delay from e‐shock onset until the sharp rise in BMA activity was quite possibly related to the lower e‐shock amplitude in second 1 (0.1 mA) compared with the amplitude in the subsequent seconds (0.15 mA). In fact, the mean BMA activity in second 1 after e‐shock onset was lower than baseline. However, in an ANOVA that focussed on seconds 1 and 2 prior to e‐shock onset and seconds 1–2 after e‐shock onset, with the latter divided into time intervals of 0.2 s, there was no interval at which BMA activity was significantly lower than baseline (*p* ≥ .10); this also applied to all the other test sessions in both experiments.

**FIGURE 4:**
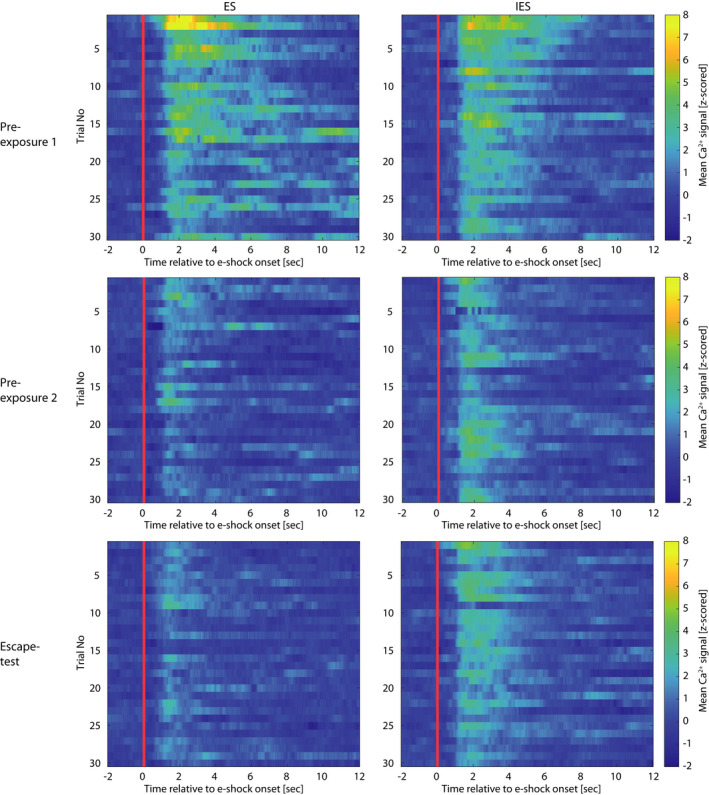
Heat maps depicting group‐mean trial‐by‐trial basomedial amygdala activity across each of pre‐exposure Sessions 1 and 2 and escape test in the learned helplessness effect experiment. Each session comprised 30 trials of either escapable or inescapable e‐shocks. Time 0 s is the time point of e‐shock onset

Aversion‐related BMA activity in ES and IES mice is presented separately for pre‐exposure Sessions 1 and 2 and the escape test in Figure 3e,f/h,g, respectively, and Figure 4. In Session 1, BMA activity was similar in IES and ES mice (*p* ≥ .12). In Session 2 (Figure 3f,h), BMA activity was higher in seconds 2–3 after e‐shock onset in IES compared with ES mice (Group × Time interaction effect: *F*_11,165_ = 2.50, *p* < .007). Also in the escape test (Figure 3g), BMA activity was higher in IES than ES mice and specifically in seconds 2–5 after e‐shock onset (Group × Time interaction effect: *F*_11,165_ = 3.05, *p* < .001). When the three phases of the LH effect paradigm were also included in the ANOVA model (i.e., Session (PE1, PE2, Escape test), Group (ES, IES), Time (seconds 1–12)), there was a significant interaction effect of Group × Time (*F*_11,510.9_ = 5.24, *p* < .0005) due to the increased BMA activity in IES mice relative to ES mice in seconds 2–5 in Session 2 and escape test. There was a trend to an interaction effect of Group × Session (*p* = .07). For the escape test, Figure 3i presents scatterplots for e‐shock transfers versus aversion‐related BMA activity to present the data for individual mice, and in addition the within‐group correlation between BMA activity and escape behaviour was analysed: for ES mice there was a significant negative correlation (Pearson's *r* = −.48, *df* = 22, *p* < .02) with higher BMA activity associated with fewer escape responses; for IES mice there was no correlation (Pearson's *r* = .00, *df* = 25, *p* = .99) with nearly all mice making a small number of escape responses. In the inter‐trial intervals, using seconds 21–35 after e‐shock onset relative to baseline, there was no effect of Time, Group or Session on BMA activity (data not shown).

Therefore, the aversion‐related behaviour of ES and IES mice developed in accordance with predictions of the LH effect paradigm. ES mice learned to make more, faster escape responses and their e‐shock motor distance increased, across the pre‐exposure sessions and escape test. In contrast, IES mice learned to make fewer transfers and their e‐shock evoked distance moved decreased in pre‐exposure sessions, resulting in a relatively low number of escape responses and low e‐shock evoked distance moved in the escape test. Concomitant with these ES‐IES behavioural differences was a relative state of high aversion‐related BMA activity in IES mice. This was the case at pre‐exposure Session 2 when e‐shock duration was equal in both groups, and particularly at the escape test when e‐shock duration was longer in IES than ES mice. Taken together, these data indicate that an uncontrollable discrete aversive stimulus (and uncertainty about its controllability as pertained for ES mice in pre‐exposure Session 1) leads to higher BMA activity than when that discrete aversive stimulus is controllable.

### 3.2 Chronic social stress and inescapable e‐shock

**FIGURE 5:**
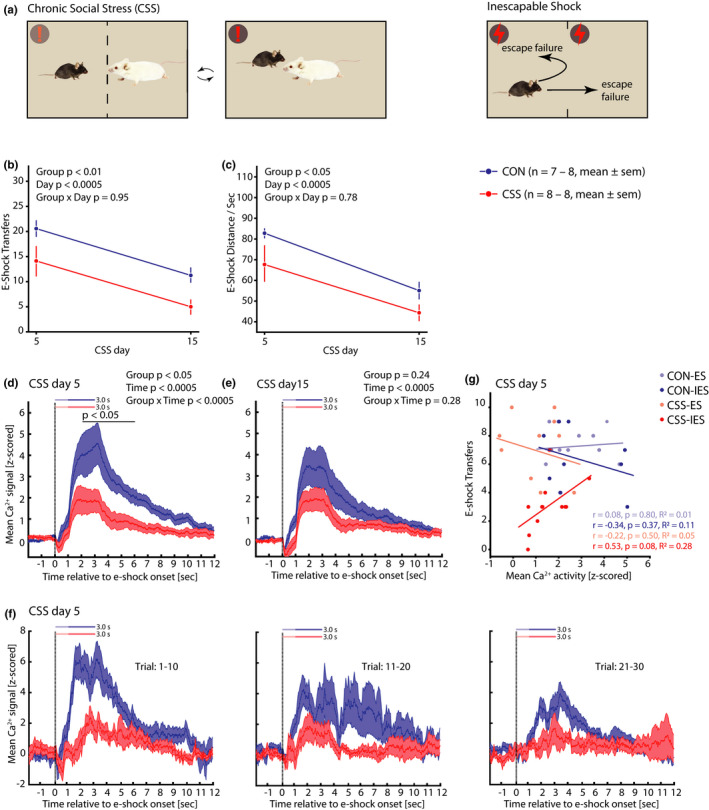
Behaviour and basomedial amygdala (BMA) activity in the chronic social stress‐inescapable e‐shock experiment. (a) Schematic for CSS and IES sessions: CSS mice were placed in the cage of a different dominant‐aggressive resident mouse on each of days 1–15 and control (CON) mice were handled each day. On days 5 and 15, each mouse was placed in the arena and received 30 × 3 s inescapable mild e‐shocks. (b) Number of e‐shock transfers by CSS and CON mice in the 30 trials on days 5 and 15, interpreted as escape attempts. (c) Mean distance moved per second of inescapable e‐shock by CSS and CON mice in the 30 trials on days 5 and 15. (d, e) Mean BMA activity in CSS and CON mice in the 30 trials on CSS/CON days 5 and 15, from 2 s pre‐ to 12 s post‐e‐shock onset. For the corresponding data expressed as ΔF/F see Figure S2. (f) In a representative CON mouse and CSS mouse (CON‐ES, CSS‐IES) on day 5, mean BMA activity in trials 1–10, 11–20, 21–30. (g) Scatterplots for e‐shock transfers versus BMA activity (mean of signal at 2–8 s after e‐shock onset) in the session on day 5 for the sub‐groups CON‐ES (*N* = 4), CON‐IES (*N* = 3), CSS‐ES (*N* = 4) and CSS‐IES (*N* = 4), with each mouse contributing data for trials 1–10, 11–20, 21–30. The regression lines are presented together with Pearson's correlation coefficient and *R* ^2^ values. For behavioural data and fibre photometry data (d, e), *p*‐values are for GLM ANOVA analysis and post hoc least significant difference testing

Following the completion of the LH effect experiment and a 30‐day interval, ES‐IES littermate pairs were allocated to either CSS or CON groups, counter‐balancing according to the number of escape responses in the escape test (*p* = .39, Figure S4a) whilst also ensuring that the e‐shock distance moved per second (*p* = .97, Figure S4b) and the escape‐test BMA Ca^2+^ activity scores (*p* ≥ .50, Figure S4c) were similar in the to‐be CON and CSS groups. The CSS and CON procedures were carried out over days 1–15 and on days 5 and 15 all mice underwent a session of exposure to inescapable e‐shocks with each e‐shock of 3 s duration (Figures 1c and 5a). For behavioural measures, Group × Day ANOVAs were conducted. With regards to e‐shock transfers (Figure 5b), mice in the CSS group made fewer transfers than did CON mice (Group main effect: *F*_1,21.5_ = 9.10, *p* < .007). In addition, both CSS mice and CON mice made fewer e‐shock transfers at day 15 compared with day 5 (Day main effect: *F*_1,21.5_ = 16.98, *p* < .0005). For e‐shock distance moved per second (Figure 5c), this was shorter in CSS mice than CON mice (Group main effect: *F*_1,22.5_ = 5.35, *p* < .04). In addition, in both CSS and CON mice, this motor response was shorter at Day 15 than Day 5 (Day main effect: *F*_1,22.5_ = 18.28, *p* < .0005). Behaviour during the ITIs is summarised in Table 1. For the measures total ITI transfers and mean locomotor distance per ITI, behaviour was similar in CSS and CON mice and remained constant across the test phases. For % time spent freezing there was a significant Group × Day interaction effect (*F*_1,17.3_ = 4.85, *p* < .05); in CSS mice % time freezing was higher at Day 15 than Day 5 (*p* < .03) whilst in CON mice it remained similar across the two time points (*p* = .52).

**FIGURE 6:**
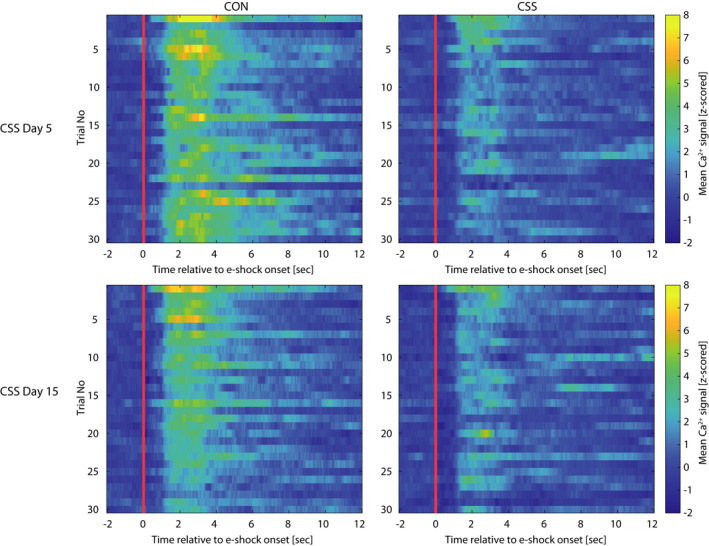
Heat maps depicting group‐mean trial‐by‐trial basomedial amygdala activity across each of the inescapable e‐shock sessions in the chronic social stress experiment. On CSS/CON day 5 and day 15, mice underwent 30 × 3 s inescapable e‐shock trials. Time 0 s is the time point of e‐shock onset

Aversion‐related BMA activity in CSS and CON mice is presented separately for Day 5 and Day 15 in Figure 5/f and e, respectively, and Figure 6. A Group × Time ANOVA was conducted for each day. On Day 5, aversion‐related BMA activity was lower in seconds 3–7 after e‐shock onset in CSS mice compared with CON mice (Group × Time interaction effect: *F*_11,142.2_ = 3.38, *p* < .0005). On Day 15, aversion‐related BMA activity did not differ significantly in CSS and CON mice (*p* ≥ .28). In an ANOVA including Day as well as Group and Time, there was a Day × Group interaction effect (*F*_1,299.0_ = 12.21, *p* < .001): this was attributable to BMA activity in CSS mice remaining similarly low on Days 5 and 15 (*p* = .72) whilst in CON mice it was higher on Day 5 than 15 (*p* < .0005). The CSS and CON groups were made up of ES and IES mice from the LH effect experiment. For Day 5, Figure 5g presents scatterplots for e‐shock transfers versus aversion‐related BMA activity to present the data for individual mice, and in addition the within‐group correlation between BMA activity and escape behaviour was analysed: In the CON sub‐groups, former ES and IES mice had a similar BMA activity and made a similar, high number of e‐shock transfers, with no significant correlation between the two measures. In the CSS sub‐groups, former ES and IES mice had a similar BMA activity and former IES mice made fewer e‐shock transfers than former ES mice; there was no significant correlation between the two measures. In the inter‐trial intervals, using seconds 21–35 after e‐shock onset relative to baseline, there was no effect of Time, Group or Day on BMA activity (data not shown).

Therefore, the difference in behaviour between CSS and CON mice in response to e‐shock was similar to that between IES and ES mice in the LH effect experiment; that is, CSS mice behaved as if they had more prior experience of IES. However, in direct contrast to the IES mice in the LH effect experiment, this behavioural state of generalised uncontrollability in CSS mice was concomitant with relatively low aversion‐related BMA activity.

## 4 DISCUSSION

The combination of valid animal models of behavioural states relevant to neuropsychiatric disorders and fibre‐photometry measurement of neural activity during these states can yield new insights into the underlying neurobiology. The present study focussed on aversion‐related BMA activity in mice during states of either specific or general uncontrollability. Compared with the many studies of the amygdala lateral and basal nuclei (e.g., Herry et al., 2008; Kim et al., 2016; Maren & Quirk, 2004; Namburi et al., 2015) and central nucleus (e.g., Tovote et al., 2016) in the context of aversion processing, the BMA has received little attention to‐date. However, it is established that the BMA contains aversion‐responsive neurons, distinct populations of these neurons are responsive to different features of aversive environments, and their activity regulates specific behaviours in aversive environments (Adhikari et al., 2015). For example, BMA “aversion neurons” fired preferentially when mice were on an open (“exposed”) arm of an elevated plus maze (EPM) or in the light (“exposed”) compartment of a light‐dark box, whereas different, BMA “reward neurons” fired preferentially when the same mice were on a closed (“safe”) arm or in the dark (“safe”) compartment. During exposure to an aversive auditory Pavlovian conditioned stimulus (CS), most of the BMA neurons responsive to it underwent inhibition of firing. In contrast, optogenetic activation of BMA neurons increased time spent on EPM open arms and decreased time spent freezing during the presentation of an aversive auditory CS (Adhikari et al., 2015). Therefore, the evidence to‐date is consistent with the BMA containing competing populations of neurons—potentially either different populations of glutamatergic principal neurons or glutamatergic principal neurons versus GABAergic interneurons—the relative firing activity of which contributes to determining the prevailing behavioural state. The present study focuses on the overall level of BMA activity in response to a specific and critical feature of aversion, namely whether it is controllable or not, and furthermore compares BMA activity during discrete and general states of aversion uncontrollability.

**FIGURE 7:**
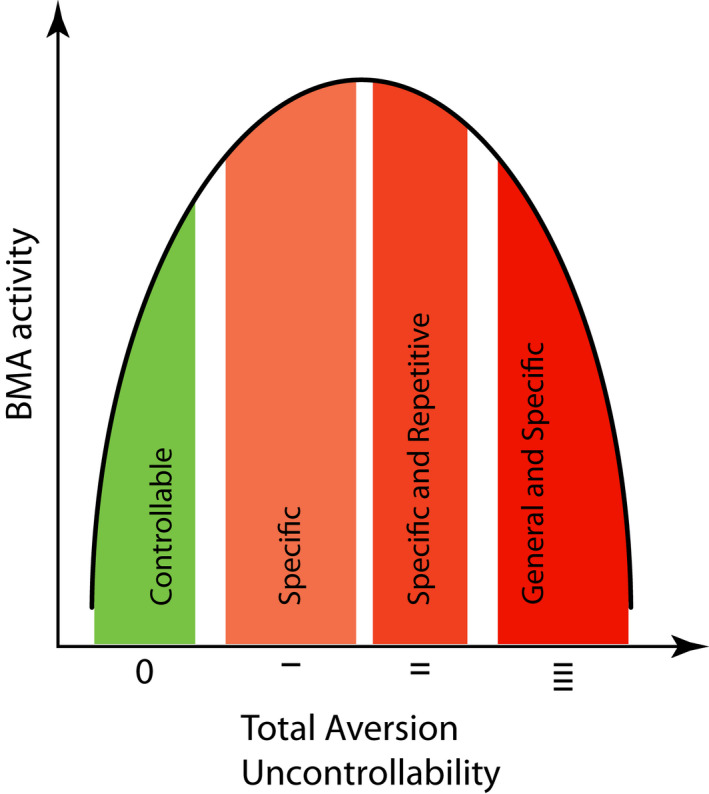
Illustration of the inverted‐U‐shaped relationship between total aversion uncontrollability and aversion‐related activity of the basomedial amygdala (BMA) as observed in the present behaviour‐fibre photometry study. In the learned helplessness effect experiment, specific uncontrollable aversion was associated with higher BMA activity than was the same specific controllable aversion. This additional BMA activity might be related to signalling and behaviour that increases the likelihood of detecting a change in aversion from uncontrollable to controllable, although the present study provides no causal evidence for this. In the chronic social stress‐inescapable aversion (CSS‐IES) experiment, general and specific uncontrollable aversion was associated with lower BMA activity than was the same specific uncontrollable aversion on its own. This attenuated BMA activity might be related to the reduced active responding to aversion (e.g., escaping e‐shock) and increased inactive responding to aversion (e.g., freezing to auditory aversive CS) reported for chronic social stress mice, and therefore a marker for generalised uncontrollability, although the present study provides no causal evidence for this. As also observed in the CSS‐IES experiment, specifically the control mice on day 15, the same specific and repetitive (un)controllable aversion can lead to some reduction in BMA activity related to that stimulus.

The first experiment investigated the effects of (un)controllability of a discrete, predictable, and mildly aversive stimulus in a specific context. Using the LH effect paradigm it has been demonstrated that mice process aversion (un)controllability (Anisman & Merali, 2001; Landgraf et al., 2015; Pryce et al., 2012) and the present behavioural findings were in full accord with the LH effect. Whilst ES mice learned that e‐shock is controllable by operant transfer responding in a two‐way escape compartment and continue to display more and more rapid transfer responses, IES mice learned that the same e‐shock is uncontrollable. IES mice displayed reduced operant responding in terms of mean locomotor distance per response, consistent with a learned motivational deficit. At the escape test, IES mice displayed a deficit in escape responding. Importantly, using the same e‐shock parameters as those used in the present study, it was demonstrated that ES and IES mice do not develop differences in pain sensitivity (Pryce et al., 2012). In pre‐exposure Session 1 (mean e‐shock of 3.4 s per trial), BMA activity was increased in seconds 2–9 after e‐shock onset, and similarly so in ES and IES mice. Therefore, in line with the single‐neuron recording data reported in Adhikari et al. (2015), the present GCaMP6 fibre photometry data indicate that BMA neuron population activity can increase in response to unconditioned aversion. In pre‐exposure Session 2, ES and IES mice received less mean e‐shock per trial (2.2 s). This was due to ES mice acquiring operant control of the e‐shock and the two groups now differed in their BMA activity: it was now higher in IES mice than ES mice, specifically in seconds 2–3 after e‐shock onset. In the escape test, BMA activity was again higher in IES than ES and now for longer—seconds 2–5—after e‐shock onset; it is important to note that e‐shock duration was now longer in IES than ES mice. Therefore, this experiment has demonstrated that BMA activity following a specific aversive stimulus is dependent on its controllability: a specific aversive stimulus of uncertain controllability or learned uncontrollability leads to higher BMA activity than does a specific controllable aversive stimulus (Figure 7). One important feature of the LH effect is that it is reversible/temporary, such that sufficient testing of IES subjects under escape test conditions results in operant learning of escape behaviour (Maier, 2001). Accordingly, the current findings suggest that high BMA activity might (a) be an adaptive response to discrete aversive environments that challenge control, and (b) contribute to the neural circuitry underlying adaptive identification of changes in controllability. In this respect, and cautioning that they do not provide evidence that changes in BMA activity cause changes in behavioural states, the current data can be integrated with the evidence that the optogenetic stimulation of the BMA artificially increases “active aversion” states such as more time on EPM open arms and decreases “inactive aversion” states such as less time freezing to an aversive auditory CS (Adhikari et al., 2015). That is, aversion‐related BMA activity might be conducive to the maintenance of active responding and particularly when the aversion is uncontrollable, which facilitates the identification of when the aversion does become controllable. Such a hypothetical neural detection system might be limited to the processing of specific aversive stimuli in an otherwise neutral environment. A to some extent similar association between neuronal population activity—also measured using GCaMP6 fibre photometry—and the specific features of the aversive environment was observed in serotonin (5‐HT) neurons in the dorsal raphe nucleus (DRN): in an active avoidance test DRN 5‐HT neuron activity decreased directly after the onset of movement (avoidance response) in response to an aversive auditory CS (specific controllable aversion), whilst during tail suspension (specific uncontrollable aversion) DRN 5‐HT neuron activity increased (Seo et al., 2019).

The second experiment investigated BMA activity in response to discrete uncontrollable e‐shock in mice also undergoing chronic and continuous exposure to social aversion. In a previous study, it was demonstrated that CSS mice made fewer escape responses to e‐shock compared with CON mice, consistent with a state of generalised uncontrollability (Azzinnari et al., 2014). In the present study, inescapable e‐shock was applied in the order that BMA activity was measured in response to exactly the same e‐shock duration in all CSS and CON mice and in both test sessions. The duration of 3 s (selected to be similar to the mean escape latency in pre‐exposure Session 1 of the LH effect experiment) was longer than that experienced by ES and IES mice in the escape test. On day 5, the relative behaviour of CSS and CON mice during inescapable e‐shock was similar to that of IES and ES mice in the LH effect experiment, respectively, with CSS mice behaving as if they had more prior experience of uncontrollability. Indeed, it was CSS‐IES mice that scored lowest on e‐shock transfers; CSS‐ES mice made a similar number of e‐shock transfers to CON mice suggesting that the procedural memory of escape behaviour was still being expressed. In contrast to IES mice in the LH effect experiment, however, CSS mice had low aversion‐related BMA activity compared with CON mice. The impact of CSS on BMA activity pertained despite some CSS mice having prior experience of controllable e‐shock and some CON mice having prior experience of uncontrollable e‐shock. This apparent paradigm shift in the relationship between aversion uncontrollability and BMA activity—IES‐induced specific uncontrollability and high BMA activity versus CSS‐induced general uncontrollability and low BMA activity—is depicted in Figure 7. On day 15 the aversion‐related BMA activity of CSS mice was no longer significantly attenuated, due to reduced BMA activity during IES in some CON mice. This suggests that for some CON mice the adaptive state of high BMA activity in response to specific but repetitive uncontrollable aversion was approaching saturation. Using single‐cell recording, some BMA neurons that responded to an auditory aversive CS did so in the form of reduced firing (Adhikari et al., 2015). Extrapolating to the current data, chronic social aversion might induce an increase in the proportion of BMA neurons that react to discrete aversion with reduced firing. It will be important to investigate whether reduced aversion‐related BMA activity is a correlate or a cause of CSS‐induced deficits in behavioural responding to environmental challenge. Related to this point, it is also important to consider the questions, does aversion (un)controllability affect BMA activity which then affects behaviour or does aversion (un)controllability affect behaviour which then affects BMA activity? The most readily apparent behavioural difference between IES and ES mice and between CSS and CON mice was motor activity. In Figure 3 it is apparent that in PE2 relative to PE1, motor activity per second of e‐shock in IES mice decreased relative to ES mice and this co‐occurred with a relative increase in their BMA activity. In Figure 5, it is apparent that at CSS/CON day 5, motor activity per second e‐shock was lower in CSS‐IES mice compared with CON‐IES mice and this co‐occurred with a lower BMA activity. The overall findings, therefore, contradict the hypothesis that aversion (un)controllability‐related differences in motor behaviour are responsible for differences in BMA activity. Of course, this lack of supportive evidence for aversion (un)controllability‐related differences in BMA activity being due to differences in behaviour does not constitute evidence that aversion (un)controllability‐related differences in BMA activity are causally responsible for differences in behaviour.

As is the case in lateral and basal amygdala nuclei, also in the BMA spiny glutamate principal neurons and GABA interneurons constitute the neuronal populations with the majority being glutamate neurons (~80%; Bloodgood et al., 2018; Duvarci & Pare, 2014). Transduction‐expression of AAV vectors driven by the hSyn promoter and administered at high titres is similar in glutamate and GABA neurons (Nathanson et al., 2009). Therefore, glutamate neurons would be expected to dominate the fibre photometry signal, such that GCaMP6 fluorescence is providing a proxy measure for the average firing state of glutamate neurons in the BMA. In CSS mice, the relative reduction in aversion‐related BMA activity might be due to decreased excitatory synaptic inputs from directly projecting neurons or increased excitatory synaptic inputs onto BMA GABA interneurons that project to neighbouring BMA glutamate neurons. Concerning afferent projection regions, Adhikari et al. (2015) used CLARITY to demonstrate that large numbers of long‐range glutamatergic projection fibres from the ventromedial prefrontal cortex (vmPFC) innervate the BMA, and both its glutamate neurons and GABA interneurons. They then applied optogenetics to either inhibit or activate the vmPFC‐BMA pathway: whilst activation increased the extinction learning of an auditory aversive CS, inhibition decreased time spent on EPM open arms (the opposite effect to direct BMA activation; Adhikari et al., 2015). Integrating these findings with the evidence that the vmPFC is a major region for processing aversion (un)controllability (Amat et al., 2005, 2008), the vmPFC‐BMA pathway is clearly a major candidate to contribute to the neural circuitries underlying CSS‐induced attenuation of aversion control, e.g., less escape/flight behaviour, and CSS‐induced accentuation of inactivity during the absence of aversion control, e.g., Pavlovian conditioned freezing, as observed in this and previous studies (Azzinnari et al., 2014; Cathomas et al., 2019; Fuertig et al., 2016; Just et al., 2018). Relevant to this, applying resting‐state fMRI in lightly anaesthetised mice, it was demonstrated that CSS led to increased resting‐state functional connectivity between PFC and amygdala (Grandjean et al., 2016). With regards to BMA output, in rats, chemical inhibition of the BMA (GABA agonist muscimol) led to the activation of the sympathetic branch of the autonomic nervous system (mean arterial pressure, heart rate) whereas its chemical activation (GABA antagonist bicuculline) reduced sympathetic activation by an unfamiliar intruder (Mesquita et al., 2016). In mice, CSS and other similar stressors lead to increased sympathetic activation, consistent with reduced BMA glutamate neuronal activity (Bergamini et al., 2018; Wohleb et al., 2011).

This study has some methodological limitations. In the second experiment, the CSS and CON mice had already experienced either ES or IES prior to the IES challenge on days 5 and 15. Although we used an interval of 30 days between the two experiments, it is likely that inter‐individual differences in e‐shock exposure in the LH effect experiment increased within‐group variance in the CSS/CON‐IES experiment. For example, the separate analysis of CSS and CON mice according to their group allocation in the LH effect experiment identified that both CSS‐IES and CSS‐ES mice contributed to the relatively low BMA activity but nonetheless differed in terms of e‐shock transfers. It will certainly be necessary to conduct a CSS‐BMA fibre photometry experiment in mice that have not had prior e‐shock exposure. Furthermore, such a study should investigate CSS effects on BMA activity to ES as well as IES, and whether one or more of optogenetic, chemogenetic or pharmacological activation of BMA activity is able to reverse CSS‐induced behavioural deficits in aversion control. Second, fibre photometry as applied in this study is not able to differentiate between different neuronal populations within the target region. Whilst the bulk of the signal is likely to have been contributed by BMA glutamate neurons, how these neurons relate to the different populations identified by Adhikari et al. (2015) is unknown. A more refined approach, potentially including fibre photometry recording from specific sub‐populations using cell type‐specific gene promoters and/or retrograde conditional virus approaches for differential expression of GCaMP6, is warranted.

## 5 CONCLUSION

Using fibre photometry to monitor e‐shock‐related BMA activity in behaving mice, the present study has demonstrated that: (a) in an otherwise non‐aversive environment, BMA activity is higher to uncontrollable than controllable aversion, and thereby potentially contributes to adaptive re‐learning when the aversion becomes controllable. (b) In a chronically aversive and uncontrollable social environment, BMA activity to an unrelated and uncontrollable aversion is reduced, and thereby possibly contributes to the maladaptive generalisation of uncontrollability. It is important to emphasise that this demonstration that BMA activity is dependent on the prevailing environmental conditions is not a demonstration that the BMA is causally involved in the neural circuitry that determines the changes in behaviour that were observed in response to these environmental conditions. Nonetheless, this study provides evidence suggesting that the BMA constitutes a neural state marker differentiating between the specific learned helplessness effect and a generalised uncontrollability state. It demonstrates the need for increased study and understanding of the role of the BMA in the pathophysiology of negative valence processing in neuropsychiatric illnesses, including major depression and post‐traumatic stress disorder.

## CONFLICT OF INTEREST

ES is an employee of TSE Systems GmbH. All other authors declare no conflict of interest.

## AUTHOR CONTRIBUTIONS

CI designed the study, conducted the experiments, analysed the data, and wrote the manuscript. AG designed the study, conducted the experiments, analysed the data, and wrote the manuscript. MB analysed the data and wrote the manuscript. HS conducted the experiments. ES designed the study. YS designed the study and wrote the manuscript. FH designed the study and wrote the manuscript. CRP designed the study and wrote the manuscript.

### Peer Review

The peer review history for this article is available at https://publons.com/publon/10.1111/ejn.15090.

## Supporting information

Fig S1‐S4

## Data Availability

Data from behaviour‐fibre photometry experiments will be made available upon request.
