# Supplementary material for: Basomedial amygdala activity in mice reflects specific and general aversion uncontrollability
Source: Eur J Neurosci. 2021 Jan 9;55(9-10):2435–54. doi: 10.1111/ejn.15090 (PMC9292353; doi:10.1111/ejn.15090)
Supplement: Supplementary file 1 — Fig S1‐S4 [file EJN-55-2435-s001.docx]

**Supplementary Information**


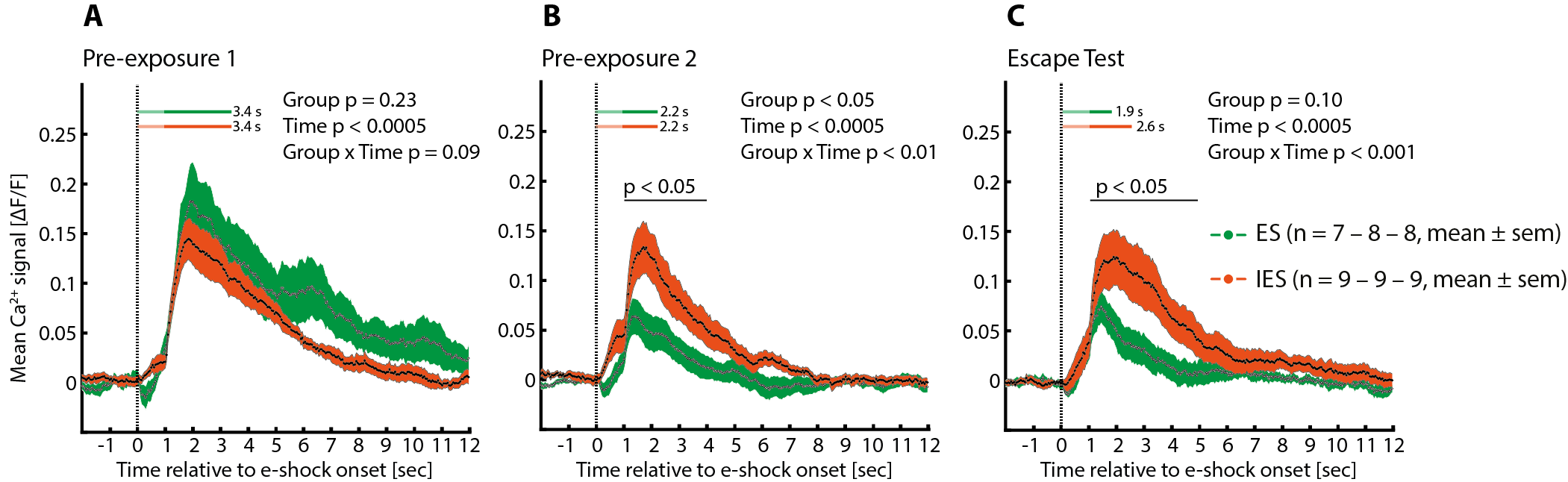


**Figure S1.** Overall mean (± SEM) BMA Ca^2+^ activity expressed as ΔF/F signals in ES and IES mice in the 3 sessions of the learned helplessness effect experiment. **A**. Pre-exposure session 1. **B**. pre-exposure session 2. **C**. Escape test. Each session comprised 30 e-shock trials. Data are from 2 s pre-e-shock to 12 s post-e-shock onset. p values are for GLM ANOVA analysis and post hoc least significant difference testing.


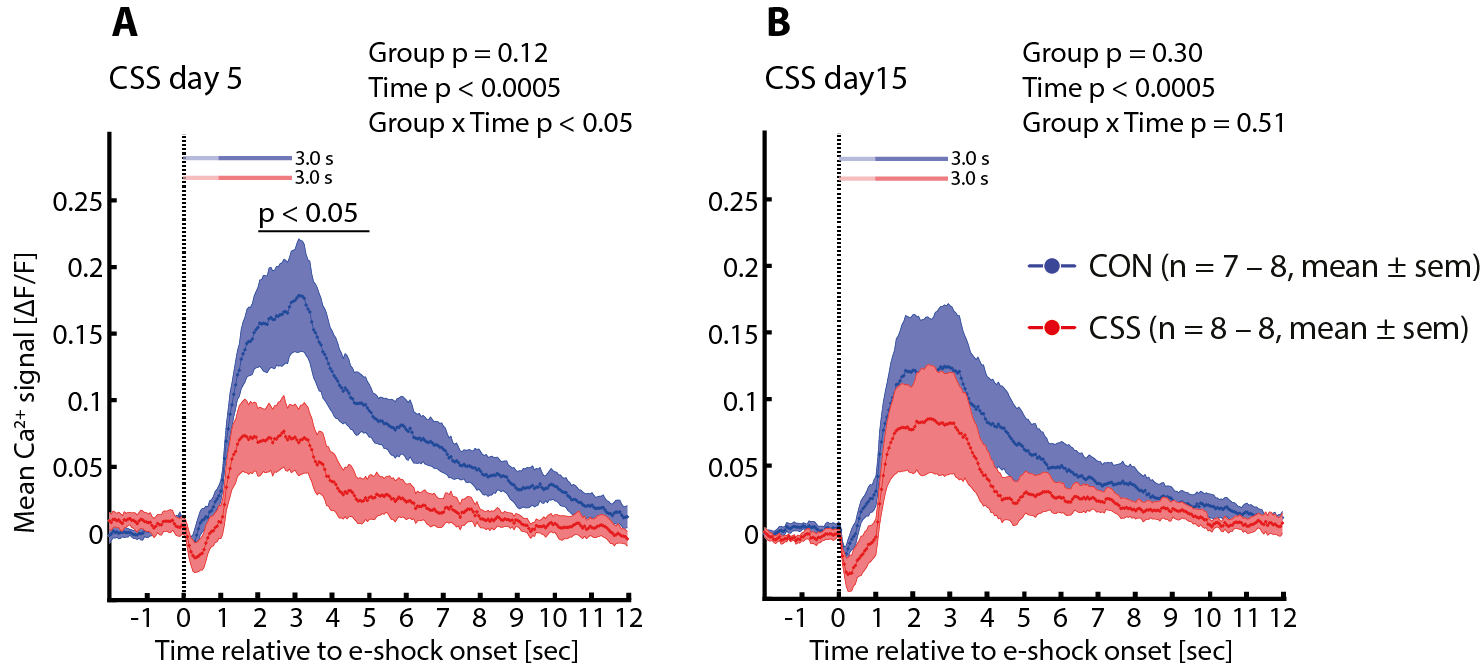


**Figure S2.** Overall mean (± SEM) BMA Ca^2+^ activity expressed as ΔF/F signals in CON and CSS mice in the 2 sessions of the chronic social stress-inescapable e-shock experiment expressed. **A**. CSS/CON Day 5. **B**. CSS/CON Day 15. Each session comprised 30 inescapable e-shock trials. Data are from 2 s pre-e-shock to 12 s post-e-shock onset. p values are for GLM ANOVA analysis and post hoc least significant difference testing.


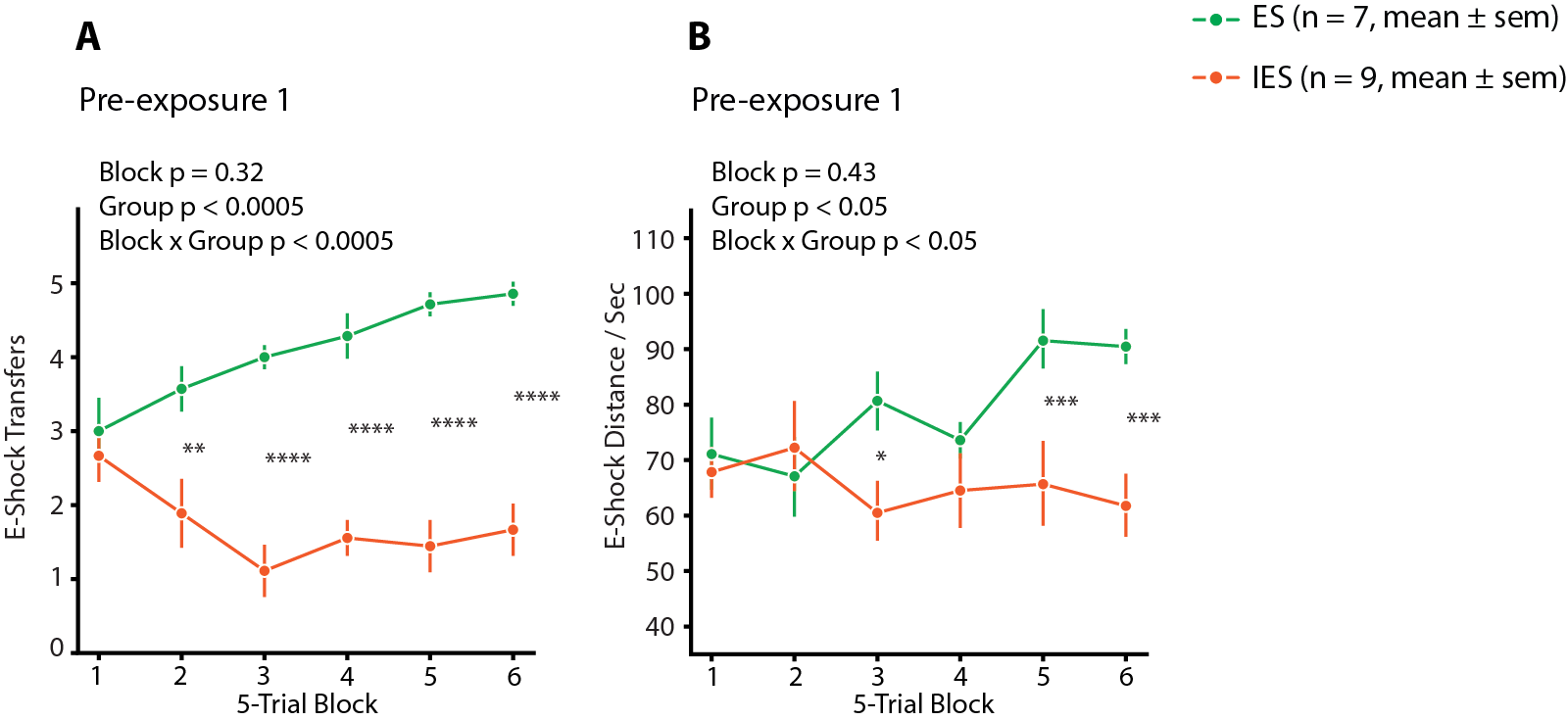


**Figure S3.** Behaviour of IES versus ES mice in the e-shock pre-exposure session 1 of the learned helplessness effect experiment presented in 5-trial blocks. **A.** Mean (± SEM) e-shock transfers; for ES mice these were escape responses whereas for IES mice they were failed “escape attempts”. Mice allocated at random to the IES and ES groups displayed a moderate and similar number of e-shock transfers in block 1 and then diverged consistently across subsequent blocks. **B.** Overall mean (± SEM) distance moved per second of e-shock exposure. Mice allocated at random to the IES and ES groups displayed a moderate and similar motor response to e-shock in blocks 1 and 2 and then diverged consistently across subsequent blocks. p values are for GLM ANOVA and post hoc least significant difference testing.


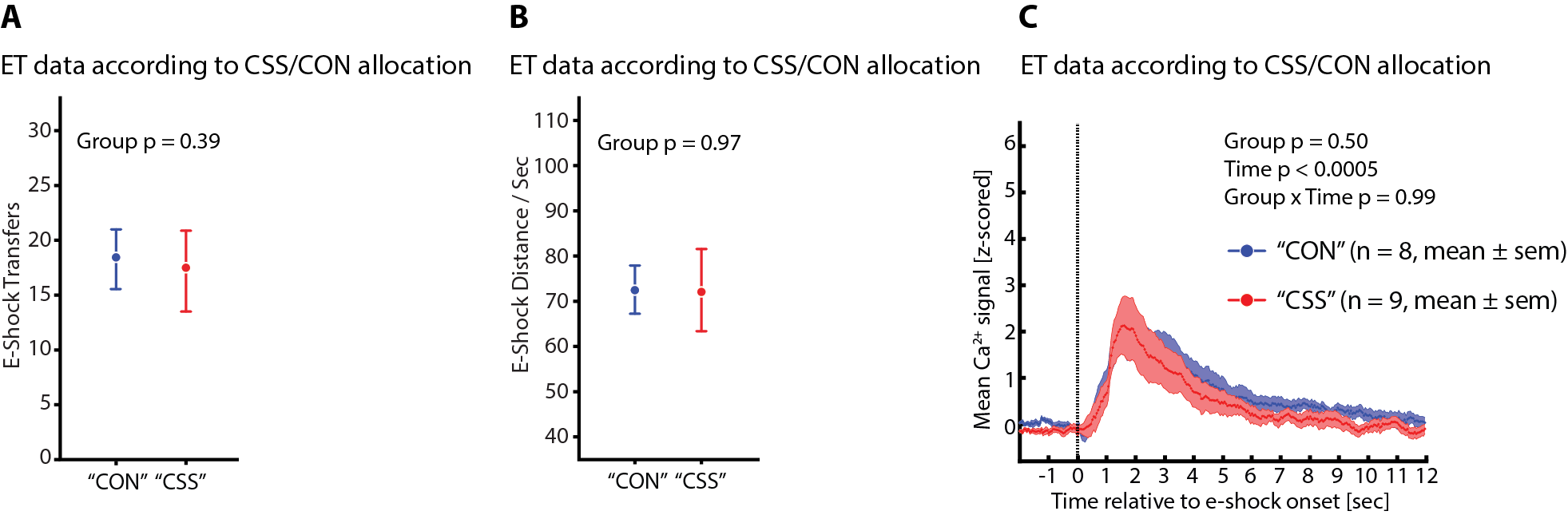


**Figure S4.** Behaviour and BMA Ca^2+^ activity of ES and IES mice in escape test of the learned helplessness effect experiment grouped according to their allocation to the control (“CON”) or chronic social stress (“CSS”) group in the subsequent CSS-IES experiment. **A.** Mean (± SEM) e-shock transfers. **B.** Overall mean (± SEM) distance moved per second of e-shock exposure. **C.** Overall mean (± SEM) BMA Ca^2+^ activity from 2 s pre- to 12 s post-e-shock onset. p values are for Student’s *t*-test or GLM ANOVA.
